# ASO Author Reflections: Value of Cytoreduction in Extensive Ovarian Cancer—Does Surgical Effort Still Matter?

**DOI:** 10.1245/s10434-019-07947-y

**Published:** 2019-10-11

**Authors:** Christina Fotopoulou

**Affiliations:** Department of Surgery and Cancer, Imperial College London and West London Gynecological Cancer Centre, Imperial College NHS Trust, London, UK

## Past

Surgery for epithelial ovarian cancer has undergone a long journey of evolution over the last decades, starting from a nihilism towards a presumed ‘hopeless’ disease over a transition of cautious speculations about the benefits of cytoreduction, to the advancement of surgical debulking techniques even outside the peritoneal cavity into the mediastinum and chest. The ‘holy grail’ of this journey has been to achieve maximal tumor clearance in an effort to derive the hoped-for therapeutic benefit. Early and continuous adopters of this radical approach have demonstrated a clear survival advantage of their patients’ populations compared with other populations where surgery was of lower effort. These pearls of wisdom and knowledge have been passed through to multiple generations of gynae-oncology surgeons in a constant strive to achieve maximal cytoreduction until, nowadays, the ‘optimal’ postoperative residual disease is accounted to be only microscopic.^1^

## Present

Debulking patients with advanced and disseminated disease has nevertheless been proven challenging and has often come at a price. Scepticists argue with increased surgical morbidity and impairment of patient’s quality of life, longer theatre times and hospital stays, and necessity of higher infrastructural and financial resources,^2^,^3^ especially for patients with higher tumor load. Under the perspective of a more adverse tumor biology that is hypothesized to mainly dictate surgical and clinical outcome independently of surgical effort, a culture of not offering surgery to patients with extensive tumor dissemination patterns has been developed, even if the disease would potentially be operable within more specialized settings. Within a population-based comparative study, Hall et al. demonstrated that also extending surgical effort to patients with higher tumor burden seems to indeed be associated with higher surgical complexity and longer theatre times, but is also independently associated with better overall survival compared with chemotherapy alone, without significant overall increase of morbidity.^2^

## Future

Through centralization of surgical care, appropriate allocation of financial and infrastructural resources, and consolidation with modern systemic-targeted agents, we aim to improve survival rates in ovarian cancer, even in those cases with more extensive tumor dissemination patterns of the disease and a seemingly less favorable tumor biology profile. Through specialized surgical training and the development of algorithms for identification of the appropriate surgical candidates, we will direct radicality towards those patients who will benefit the most, and improve not just the surgical but also the overall clinical outcomes.^4^,^5^ It is prime time that the major systemic advances are being paired and complemented with an equivalently high surgical effort, all under the umbrella of personalized surgical care.

## References

[1] Schorge JO, Bregar AJ, Durfee J, Berkowitz RS (2018). Meigs to modern times: the evolution of debulking surgery in advanced ovarian cancer. Gynecol Oncol.

[2] Hall M, Savvatis K, Nixon K (2019). Maximal-effort cytoreductive surgery for ovarian cancer patients with a high tumor burden: variations in practice and impact on outcome. Ann Surg Oncol.

[3] Aletti GD, Podratz KC, Moriarty JP, Cliby WA, Long KH (2009). Aggressive and complex surgery for advanced ovarian cancer: an economic analysis. Gynecol Oncol.

[4] Tseng JH, Cowan RA, Zhou Q (2018). Continuous improvement in primary debulking surgery for advanced ovarian cancer: do increased complete gross resection rates independently lead to increased progression-free and overall survival?. Gynecol Oncol.

[5] Aletti GD, Dowdy SC, Gostout BS (2009). Quality improvement in the surgical approach to advanced ovarian cancer: the Mayo Clinic experience. J Am Coll Surg.

